# The nonlinearity of pupil diameter fluctuations in an insight task as criteria for detecting children who solve the problem from those who do not

**DOI:** 10.3389/fpsyg.2023.1129355

**Published:** 2023-06-23

**Authors:** Sebastián Vásquez-Pinto, Diego Morales-Bader, Ralf F. A. Cox, Felipe Munoz-Rubke, Ramón D. Castillo

**Affiliations:** ^1^Centro de Investigación en Ciencias Cognitivas, Facultad de Psicología, Universidad de Talca, Talca, Chile; ^2^Facultad de Ingeniería y Ciencias, Universidad Adolfo Ibáñez, Santiago, Chile; ^3^Department of Developmental Psychology, Faculty of Behavioural and Social Sciences, Heymans Institute for Psychological Research, University of Groningen, Groningen, Netherlands; ^4^Instituto de Psicología, Universidad Austral de Chile, Puerto Montt, Chile

**Keywords:** insight problem solving, entropy, fractal scaling, self-organization, pupil diameter fluctuations, 8-coin task

## Abstract

Insights, characterized by sudden discoveries following unsuccessful problem-solving attempts, are fascinating phenomena. Dynamic systems perspectives argue that insight arises from self-organizing perceptual and motor processes. Entropy and fractal scaling are potential markers for emerging new and effective solutions. This study investigated whether specific features associated with self-organization in dynamical systems can distinguish between individuals who succeed and those who fail in solving insight tasks. To achieve this, we analyzed pupillary diameter fluctuations of children aged 6 to 12 during the 8-coin task, a well-established insight task. The participants were divided into two groups: successful (*n* = 24) and unsuccessful (*n* = 43) task completion. Entropy, determinism, recurrence ratio, and the β scaling exponent were estimated using Recurrence Quantification and Power Spectrum Density analyses. The results indicated that the solver group exhibited more significant uncertainty and lower predictability in pupillary diameter fluctuations before finding the solution. Recurrence Quantification Analysis revealed changes that went unnoticed by mean and standard deviation measures. However, the β scaling exponent did not differentiate between the two groups. These findings suggest that entropy and determinism in pupillary diameter fluctuations can identify early differences in problem-solving success. Further research is needed to determine the exclusive role of perceptual and motor activity in generating insights and investigate these results’ generalizability to other tasks and populations.

## Introduction

Insights are an intriguing phenomenon in problem-solving research. Insights occur when people suddenly discover the only possible solution after unsuccessfully trying to solve a problem while expressing astonishment (Chronicle et al., 2004; Chu and MacGregor, 2011; Weisberg, 2015). So far, theoretical approaches that account for the occurrence of insights propose unconscious or automatic restructuring mechanisms and representations that operate through symbolic or algorithmic systems assembled to reduce error (Ohlsson, 1992, 2011; Knoblich et al., 1999).

According to these approaches, the inadequate representation of a problem creates a space of solutions that cannot solve the task (Knoblich et al., 1999). As a result, the repeated unsuccessful attempts create stagnation and the feeling that all possible solutions have been exhausted (Impasse; Ohlsson, 1992). One theory that integrates these elements is the Representational Change Theory (RCT), which argues that insight involves the conceptual and perceptual reformulation of the problem. Even when insight tasks may appear to require only a few simple steps for their solution, the real solution can be challenging to discover. According to RCT, incorrect representations of the problem can prevent us from identifying an efficient strategy and executing the correct actions. In order to overcome this difficulty, it is necessary to reorganize the problem representation using two mechanisms: relaxing self-imposed restrictions (restriction relaxation) and breaking down the problem into simpler components that are easier to manage perceptually (chunk decomposition). By utilizing both of these mechanisms, the solver can increase the chance of finding the solution to the problem (Jones, 2003; Öllinger et al., 2013). This process takes place outside of a person’s consciousness, displaying a new space of solutions, and is often accompanied by positive emotions such as pleasure, surprise, and certainty, among others (Danek et al., 2014; Shen et al., 2016). It is important to note that RCT appeals to the use of symbols and representations and implicitly assumes top-down processes to explain the phenomenon of insight (for an alternative view, see Satisfactory Progress Theory; MacGregor et al., 2001; Ormerod et al., 2002).

In contrast, the group of Dixon and collaborators sought to approach insight from a perspective linked to dynamic systems. This view addresses several criticisms raised against the traditional cognitive model, such as the rigidity of the concept of representation and the model’s inability to account for the spontaneous emergence of new strategies or new patterns of response (Stephen and Dixon, 2009). According to this view, the mechanisms involved in insight are of a perceptual and motor nature. This approach offers an ecological view in which cognition is context-dependent. In that sense, Dixon and collaborators proposed that insight is a self-organizing behavior that emerges from the coupling of perceptual-motor processes during phase transitions (Dixon and Bangert, 2002; Dixon and Dohn, 2003; Stephen et al., 2009; Stephen and Dixon, 2009; Dixon et al., 2010). Self-organization refers to the emergence of a new structure or pattern where the components, by themselves, cannot explain what emerges; and where there is no direction from either a central executive or an external agent (Ashby, 1962; Kelso, 1995; van Orden et al., 2003; Smith, 2005; Castillo et al., 2010).

### Dynamical features related to insight

According to Dixon and his colleagues, behavior, and cognition continuously self-organize themselves based on the fluctuations of information and the uncertainty of the environment (Kugler and Turvey, 1988; Bak, 1996; Jensen, 1998; van Orden et al., 2003; Holden, 2005). This conjecture emerged after they observed two features in the dynamics of behavior linked to self-organizing physical and biological systems: a peak in entropy and a sharp manifestation of 1/f noise, both just before discovering a new and effective solution. These characteristics indicate the dynamic behavior of some complex systems as they move from one state to another (Kelso, 1995). 1/f noise, also known as pink noise, has a power spectrum proportional to the inverse of frequency and is often observed in systems that exhibit long-range correlations or self-similarity (Holden, 2005).

Dixon and Bangert (2002) reported that the emergence of insights in participants during the Gear System Task experiment was due to self-organizing patterns of behavior that emerged to dissipate entropy. The new strategy was the product of the interaction between the participant’s perceptual-motor system and the environment through force tracing, which introduced entropy. Entropy measures the amount of uncertainty or randomness in a signal. The more uncertain or random the signal is, the higher its entropy will be. Conversely, if the signal is predictable or orderly, its informational entropy will be lower. According to Dixon and collaborators, the cognitive system that is compelled to dissipate such entropy generates a new organization or structure that is more efficient in dissipative work (van Orden et al., 2003).

The transition from the force-tracing strategy to the alternation strategy was found in preschoolers, schoolers, young people, and adults (Dixon and Bangert, 2002; Dixon and Dohn, 2003). In these groups, the angular velocity of finger movements showed an abrupt increase and decrease in entropy before discovering the alternation strategy through the tracing strategy. Furthermore, the angular velocity adopted the shape of a power-law distribution, which is an asymmetrical distribution that shows the inverse relationship between the frequency of a phenomenon and its magnitude on a logarithmic scale (Stephen and Dixon, 2009). Dixon conducted other experiments in which uncertainty (randomness) was intentionally introduced to the gear-related tasks. As a result, more people could find a solution, and they did so more quickly. In the cases where the task was solved, sudden changes in the entropy of the angular velocity were observed. Furthermore, when analyzing the distribution of eye fixations, the results adopted the form of a power law distribution.

Power law distributions are the distribution of objects known as fractals, which themselves are recursive, self-similar patterns at different scales of observation with no mathematical end. In practical terms, fractals reproduce the same variability on different time scales. In other words, they are objects reproduced on more minor scales and where their relative size is inversely related to their frequency (Brown and Liebovitch, 2010). This type of invariant relation between size and frequency has been detected in various tasks such as time estimations, mental rotation, lexical decision, rhythmical aiming, motor-control tasks, perception of reversible figures, visual search and visual matching, implicit measures of stereotyping, prejudice, self-esteem and physical self-perception reports (Gilden, 2001; Aks and Sprott, 2003; Delignières et al., 2004; Correll, 2008; Wijnants et al., 2009, 2012a; Diniz et al., 2011; Castillo et al., 2015).

While 1/f-pink noise is ubiquitous in many complex systems, it is not a universal feature of all signals. For example, pupil diameter fluctuations over time cannot behave like 1/f noise because the pupil does not expand and contract rapidly and sharply under normal conditions. Instead, these fluctuations are better described by Brownian noise, which resembles a random walk process characterized by small and gradual changes in size. There is compelling evidence that this type of signal exhibits Brownian noise instead of 1/f or pink noise (Moon et al., 2014; Kim and Yang, 2017). Since these two signals differ radically, the question remains whether, with Brownian noise time series, it is possible to detect significant differences such as those found in the studies mentioned above.

In summary, Dixon and colleagues proposed that behavior and cognition self-organize themselves based on the fluctuations of information and the uncertainty of the environment. This self-organizing process involves dissipating entropy to generate a new organization or structure that is more efficient in dissipative work. The emergence of insights is due to self-organizing patterns of behavior that appear to dissipate entropy and is characterized by a peak in entropy and a sharp manifestation of 1/f noise. These features indicate the dynamic behavior of some complex systems as they move from one state to another and are observed in different cognitive and behavioral tasks. It is important to note that Dixon and his colleagues did not study dilated pupils. In contrast, other researchers who have analyzed fluctuations in pupillary diameter have observed that this signal produces a noise that can be classified as Brownian noise.

### The gear system task as a pseudo-insight task and the 8-coins task as an insight task

Even though human behavior expresses these two features detected in complex dynamic systems during the resolution of the Gear system task, these results have not been immune to criticism. One important criticism is that the Gear System task lacks the characteristics of a real insight problem (Chronicle et al., 2004; Chu and MacGregor, 2011). The main difference between insight problems and other types of problems (e.g., analytical problems) is a phase known as an impasse. Although it is a matter of discussion (Fleck and Weisberg, 2013; Danek et al., 2014; Webb et al., 2016), several models point to the existence of a period of stagnation that precedes the occurrence of an insight (Wallas, 1926; Ohlsson, 1992; Öllinger et al., 2014). Misrepresenting a problem would generate that impasse, usually associated with negative emotions (Fleck and Weisberg, 2004). The impasse is reflected in a cessation of the resolution activity and by repeating unsuccessful attempts (Beeftink et al., 2008). Because of this, solvers think they have tried all the solutions unsuccessfully. After that, a true insight, the only possible solution emerges abruptly due to a mental reorganization of the problem (Knoblich et al., 1999; Öllinger et al., 2013). In the Gear System task, no impasse occurs because, from the beginning, participants can solve the problem without significant difficulties, and different strategies might coexist without any mental reorganization. For this reason, the Gear System task is classified as a pseudo-insight task, limiting the applicability of Dixon’s findings to real insight tasks.

Given this criticism, in the current study, we explored whether the findings of Dixon and colleagues are generalizable to real insight tasks. More specifically, we asked whether it is possible from entropy fluctuations and fractal scaling to differentiate children who solve an insight problem from those who do not. For this purpose, we used the 8-coins task, considered a real insight problem task (Ormerod et al., 2002; Gilhooly et al., 2010; Öllinger et al., 2013). This task consists of presenting eight black coins, which participants must reorganize with just two moves such that each coin touches three other coins. The solution involves overlapping two key pieces into two separate groups of three pieces each.

The 8-coin task was thoroughly studied by Öllinger et al. (2013), who manipulated the presence/absence of certain perceptual cues in the task. For example, in some conditions, the eight coins were located so that all touched each other (grouped) or only touched some other (ungrouped). Moreover, other conditions had one coin positioned on top of another (3D cue) or not (2D cue). These cues affected participants’ performance, showing higher solution rates in ungrouped 3D configurations than in grouped 2D configurations (Öllinger et al., 2013).

Although the 8-coin task is a simple problem, the solution can be difficult for individuals. According to the Representational Change Theory (RCT), this would occur because an erroneous representation of the problem would prevent participants from performing 3D movements and thinking about strategies that involve separating the initial group of pieces. Hence, insight would appear as a product of the reorganization of said representation in terms of two mechanisms: restriction relaxation and chunk decomposition (Öllinger et al., 2013). The first is the solver’s relaxation of self-imposed restrictions (e.g., only performing 2D movements). The second would be to decompose the problem into simpler components, more manageable units perceptually (e.g., to split the eight-coin group into two four-coin groups). The deployment of both mechanisms would allow the participants to find the solution.

### Pupillary diameter fluctuations as a signal closer to the insight phenomena

To study insight in the 8-coin task, we focused on a physiological signal that may be related to this event. We analyzed the pupillary diameter fluctuations of participants. It is well-known that the pupil constricts or dilates by reflex in response to environmental stimuli. For example, it contracts to luminous stimuli (Pupillary Light Response or PLR), or it constricts to a stimulus presented near fixation (Pupillary Near Response or PNR) (Mathôt, 2018). However, it is less known that the pupil diameter fluctuates according to the Psychosensory Pupil Response (PPR), in which pupil dilation is associated with cognitive activities such as mental effort (Hess and Polt, 1960, 1964); experience of surprise (Friedman et al., 1973; Preuschoff et al., 2011), disambiguation of stimuli (Suzuki et al., 2018) and the change in expectation (Gilzenrat et al., 2010); core components of the insight experience (Chu and MacGregor, 2011). Moreover, a study demonstrated that increases in pupil diameter were associated with insight achievement, both in the form of “false insight” and “real insight” (Salvi et al., 2020). These authors registered the participants’ oculomotor activity while participants solved a battery of problems and discovered that microsaccades were related to resolution through mental analysis. Additionally, they found that increases in pupil diameter were related to problem-solving through insight (Salvi et al., 2020).

### Recurrence quantification analysis (RQA) and power spectrum density (PSD) applied to insight phenomena

Based on the analyses conducted by Dixon and colleagues, we utilized two statistical techniques to examine pupil diameter fluctuations: Power Spectrum Density (PSD) for estimating power-law scaling (Hausdorff et al., 1995; Peng et al., 1995; Holden, 2005; Kello et al., 2007; Wijnants et al. 2012a,b); and Recurrence Quantification Analysis (RQA) for estimating entropy, recurrence ratio, and determinism (Marwan and Kurths, 2002; Shockley, 2005; Orsucci et al., 2006; Marwan et al. 2007; Bastos and Caiado, 2010; Castillo et al., 2015; Ngamga et al., 2016; Lira-Palma et al., 2018; Morales-Bader et al., 2023).

**Figure 1:**
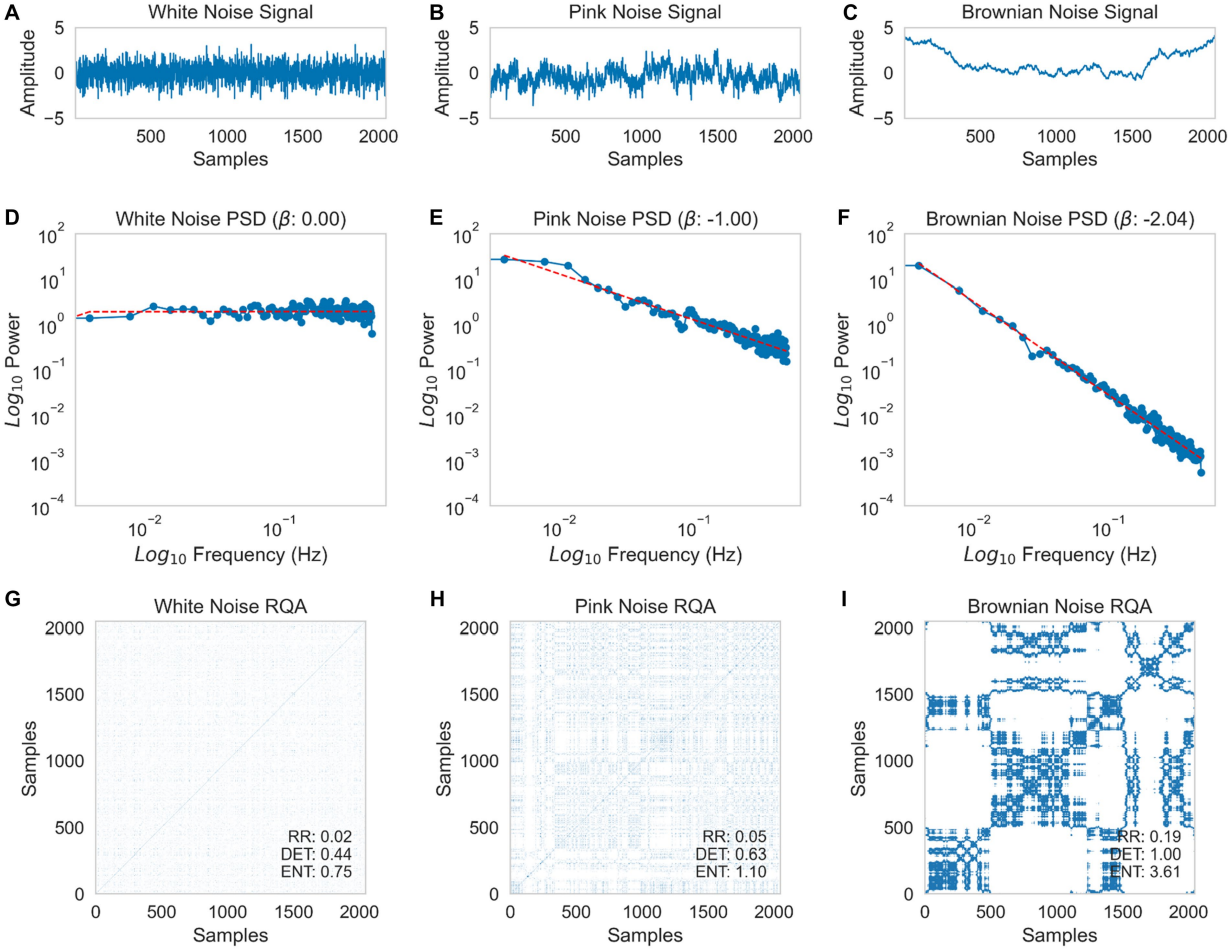
Time-series simulation of white noise **(A)**, pink noise **(B)**, and Brownian noise **(C)** with 2048 samples. Estimation of Power Spectral Density (PSD) using the Welch method for white noise **(D)**, pink noise **(E)**, and Brownian noise **(F)**. Recurrence Plot of the Recurrence Quantification Analysis (RQA) for white noise **(G)**, pink noise **(H)**, and Brownian noise **(I)**. For the RQA, a delay of 1, embedding dimensions of 3, and a threshold of 0.6 were used.

PSD is a method of estimating the power spectrum of a signal from its time-domain representation, which characterizes its frequency content and helps to identify periodicities (Kello et al., 2007). This method decomposes the signal into different frequencies using a Fourier transformation. Low-frequency sine waves represent slow changes, high-frequency waves represent fast changes, high-amplitude sine waves represent large changes, and low-amplitude sine waves represent small changes. By plotting the frequency and power of each sine wave on log/log scales, the paired amplitudes and frequencies exhibit a proportional relationship, forming a scaling relation. The slope of the regression line provides a rough estimate of the 1/f scaling exponent (Castillo et al., 2010). By analyzing the slope of the scaling exponent of the power spectral analysis, the Beta (𝛃) scaling exponents of the simulated time series of White, Pink (1/f), and Brownian noise can be estimated (Figures 1A–C, respectively). Panels D, E, and F depict three logarithmic scale plots in which the time series have been submitted to Power Spectral Analysis. The white noise series has a slope of 0, the pink noise series has a slope of −1, and the Brownian noise series has a slope of −2.04 (𝛃 ≈ −2). These results are presented in logarithmic scale plots.

RQA is a non-linear, multidimensional statistical analysis used to discover attractors in time series whose signal is irregular, multidimensional, and non-stationary (Riley and Turvey, 2002; Marwan et al., 2007; Wijnants et al., 2012b). RQA has the advantage that from a single time series subjected to a time-delay treatment, it is possible to estimate the value of other variables to reconstruct the dynamic behavior of a system (Takens, 1981). RQA makes no assumptions about the distribution or size of the data. Measures extracted with RQA are estimated from recurrence plots (RPs), which are graphical representations of a matrix of recurrence that highlights aspects that cannot be detected in the original data set. This matrix is the autocorrelation of the same signal with some delay. Figures 1G–I show three Recurrence Plots (RPs). These plots represent each time series with itself (on the *X* and *Y* axis) (Webber and Zbilut, 2005). With this procedure, it is possible to identify points where the series tends to return (recurrence), informing the “preference” of the systems to state in a specific trajectory. Several quantitative and reliable measures can be estimated from RPs, such as the degree of the system’s entropy (ENT), the recurrence ratio (RR), and the proportion of determinism (DET) (Marwan et al., 2007). ENT estimates the degree of disorder expressed by a system based on the Shannon equation. Mathematically, ENT is the probability distribution of the diagonal line lengths. RR represents the likelihood of a particular state recurring. Finally, DET is related to the system’s predictability (Stephen and Dixon, 2009). RR is mathematically defined as the density of recurrence points in a plot. DET is calculated as the percentage of recurrence points that form diagonal lines in the recurrence plot with the minimum length.

The white noise series (Figure 1G) is characterized by a system that moves randomly without any preferred trajectory, resulting in the absence of recurrences. On the other hand, the 1/f-pink noise (Figure 1H) and Brownian noise series (Figure 1I) demonstrate a preference for specific trajectories, which can be observed as marked patterns of recurrences. To establish the degree of entropy and other derived measures, the observed pattern in the RP must undergo a series of analyses. However, before implementing these calculations, three parameters must be estimated: the delay, embedded dimensions, and the radius (Shockley, 2005). For the simulated 2048-point time series of White, Pink (1/f), and Brownian noises, these parameters were 1, 3, and 0.6, respectively. The values obtained from the Recurrence Plots (RPs) exhibit differences in entropy, recurrence ratio, and determinism. The simulated series of White noise shows the lowest values, pink noise shows intermediate values, and Brownian noise has the highest values. White noise is the most random, with a low preference for a particular trajectory in phase space (RR) and a low ability of the initial states to predict future states (DET). On the other extreme, Brownian noise displays a higher level of order or structure, with a marked preference for a particular trajectory in phase space and high levels of predictability due to the semi-periodic nature of the system (Marwan et al., 2007).

In sum, the present study aimed to test Dixon’s hypotheses on the 8-coins task, which has a unique correct solution and no alternative strategies. We analyzed participants’ pupillary fluctuations as a physiological signal to study differences between subjects who solved and did not solve the task. Given that visual neurocognitive systems modulate pupillary fluctuations, we hypothesized that children who solved the task would exhibit entropy changes just before finding the solution. This pattern would be absent in those who did not solve the task. Additionally, as previously reported by Dixon and colleagues, successful problem-solvers would exhibit a fractal structure similar to 1/f-pink noise. However, previous studies on pupillary dilation during cognitive tasks suggest that the temporal structure should be closer to Brownian noise. Therefore, by exploring these different possibilities of temporal structures, we aim to shed light on pupillary fluctuations and their relationship to problem-solving cognitive processes.

## Method

### Participants

**Figure 2:**
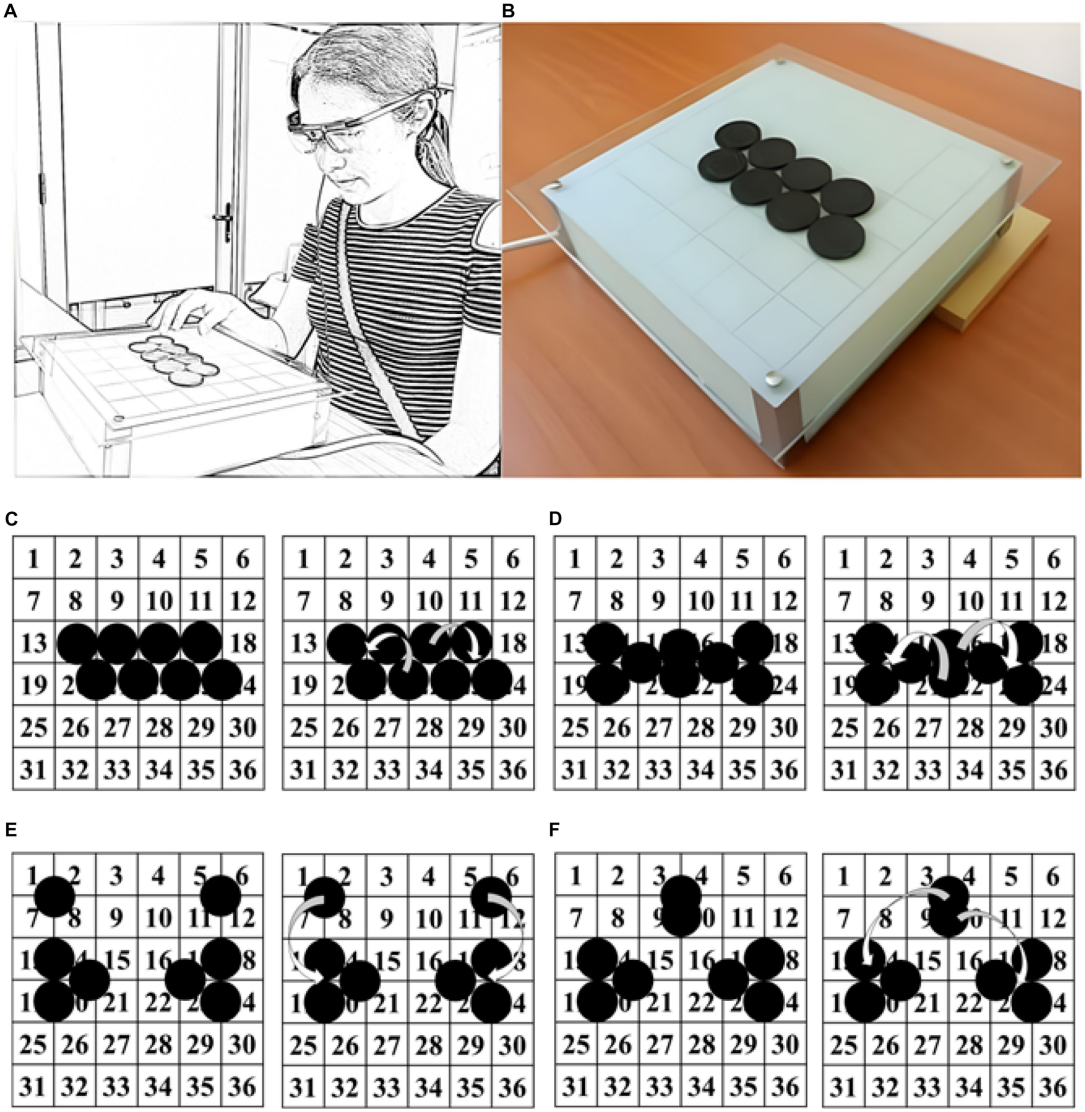
**(A)** Sketched picture of a participant using eye-tracking glasses (Tobii Eye Tracker X2-60) during the insight problem-solving task. **(B)** Picture of the grouped coins with 2D cues distributed on the platform. **(C)** At right, configuration with grouped coins and 2D cue. **(D)** Configuration with grouped coins and 3D cue. **(E)** Configuration with ungrouped coins and 2D cue. **(F)** Configuration with ungrouped coins and 3D cue. In all four configurations, at the right, the arrows indicate the coins that must be moved and where they must be placed to solve the task successfully.

We recruited 76 children between the ages of 6 and 12 (*M* = 9.39; SD = 1.53) from public schools in Talca City. However, we excluded nine participants from the sample because they solved the task too quickly or withdrew from the study within 120 s of starting the task. Before starting the experiment, we obtained informed consent from the parents or caregivers (IRB FONDECYT 1161533) and provided an informed assent document to the children. The experiment only began once the children explicitly agreed to participate. Our final sample consisted of 67 children (36 boys, 31 girls) randomly assigned to the four experimental conditions (See Figure 2). No significant differences in average age across the four conditions were found [*F* (3, 63) = 0.914, *p* = 0.439].

We used G-Power to estimate the required sample size (Faul et al., 2007). We employed a 2 × 2 experimental design, which included four groups (3D vs. 2D x grouped vs. ungrouped). Each group was measured 56 times (windows) on their dependent variables, with an effect size of 0.30, an alpha error of 0.05, and a power of 0.80. The total number of participants suggested was 64 (16 participants per group). Subsequently, we estimated the post-hoc sample size required to compare two groups of children (non-solvers and solvers) using an effect size of 0.30, an alpha error of 0.05, a power of 0.80, and 56 measurements of their dependent variables. The estimated sample revealed that we needed 20 children (10 per group) to achieve the desired statistical power.

### Materials and methods

We used the 8-coin task as an insight task (Öllinger et al., 2013). This task contained eight circular pieces (or “coins”) of 40 mm in diameter and 2 mm high. These were placed on a 7 cm x 31 cm x 31 cm (“ABITARE, LIGHTING,” 220v) platform. The surface was divided into 36 cells of 4 cm2 each (Figure 2B). One of four possible 8-coin configurations was presented (Figure 2, Panels C to F) with approximately 10 degrees of inclination relative to the participant. These four configurations resulted from combining two factors with two levels each (grouping and dimensionality). Grouping refers to configurations where the eight coins could be grouped in one big segment (Panel C and D) or separated into subsets (Panel E and F). Dimensionality refers to configurations that could present a 3D cue (coin overlapping other; Panel D and F) or where this cue is absent (Panel C and E). These factors combined formed four conditions: Grouped-2D, Ungrouped-3D, Grouped-3D, and Ungrouped-3D. An eye-tracking system was used to record eye activity (Tobii Eye Tracker X2-60; Figure 2A). This system consists of glasses that record eye movements (i.e., fixations, attention, and pupil behavior) and a tablet that receives and records this information in real-time. From each child’s task recording, a video of his/her eye movements was obtained by the Tobii Studio software.

### Procedure

Each participant was evaluated in an isolated laboratory room with two experimenters (E1 and E2). E1 (located next to the child) was in charge of positioning the eye-tracking glasses, giving instructions, rearranging the pieces whenever an attempt was unsuccessful, and providing feedback (i.e., briefly explaining why the attempt did not accomplish the solution). E2 (located behind the child) was responsible for recording a video with the information captured by the eye-tracking glasses and monitoring that each child was looking at the board when solving the task. Once the eye-tracking glasses were calibrated, the experiment began with E2 starting the recording and E1 reading the following instruction:

“In this task, there are 8 circular coins. Please, count them: one, two, three… and eight coins. As you can see, all coins are identical. Your task is to move only two coins so that each coin touches the other three coins. Note how they are distributed on the platform; some touch two other coins, some touch three, and so on. You can move any coin you want. Remember that your task is to move only two coins, so each coin touches another three coins. You must move the coins whenever you have a solution in mind. Remember that you can move two coins per attempt. You must inform the experimenter when you think you have found the solution”.

After each unsuccessful attempt, participants were instructed to avert their gaze from the platform and look in a different direction. At this moment, E1 resets the coins to their initial configuration and begins the next attempt. This procedure was repeated until the task was solved, or until a minimum of 20 min had passed (after which participants had the option to discontinue), or a maximum of 40 min. If the children had not found the solution after 20 min, once they had completed the attempt, they were asked if they wanted to continue. If they decided to continue, the task continued for 20 more minutes. The time range of 20 to 40 min was established based on a pilot sample in which coins of various sizes and materials (metal, wood, and poker chips) were tested on different surfaces. While this time frame was initially determined preliminarily, it was consistently applied to all children in the study. It should be noted that there were no instances of children voluntarily giving up before the 20-min mark, nor were there any cases of children wanting to continue beyond the 40-min limit if they had not yet solved the problem. Regardless of the result, each child received a didactic toy as a reward for their participation.

### Data processing

**Figure 3:**
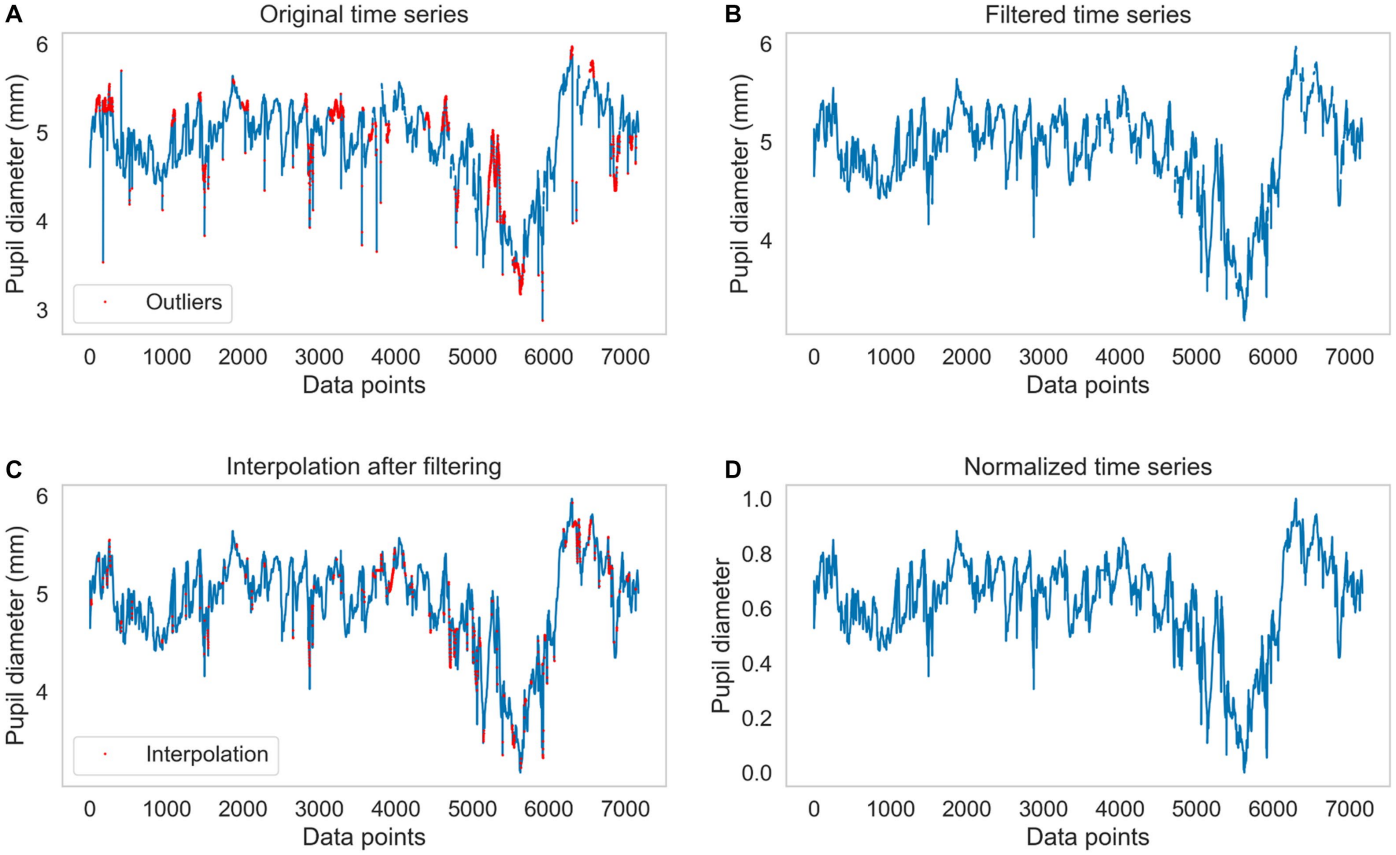
Example of data preprocessing. **(A)** Shows the original raw data. The red dots represent the outliers detected by the Hampel filter. **(B)** Shows the data after applying the filter. **(C)** Shows the series with the missing data interpolated. The red dots correspond to the interpolated points. **(D)** shows the normalized time series.

Pupil diameter fluctuations were extracted from the eye with the most information from each participant. A Hampel Filter was applied to remove atypical data (records with pupil diameters ±3 SD). Then some data lost due to blinking or loss of eye tracking were interpolated with the Akima interpolation method, as one of the suggestions of Dan et al. (2020) for this type of data. When the window of missing values was equal to or less than 2 s (100 data points), an interpolation was performed; otherwise, those missing values were assumed missing and deleted. Finally, the data were normalized using a MinMax normalization between 0 and 1 to compare subjects with different pupil sizes on the same scale. Figure 3 shows the data preprocessing at each step.

## Data analysis

### RQA and PSD analyses

A windowed recurrence quantification analysis (RQA) was performed. Each window comprised 500 data points, corresponding to approximately 100 s of the task. The windows were moved in steps of 100 data points in each step. For example, the first window went from 0 to 500 data points, the second from 100 to 600, and so on. Since subjects took different times to finish the task or give up, to analyze equivalent time instants and compare the same moments between subjects, we took the last 120 s of each subject. These last 120 s are equivalent to the last 56 windows of the RQA. The Python library PyRQA (Rawald et al., 2017) was used to perform the RQA.

The RQA has three main parameters to be estimated. The first is the lag, estimated with the average mutual information (AMI) technique, selecting the lag where the function decays to 1/e of its value at zero. Since we are working with windows of 500 data points and steps of 100 data points, the lag was estimated for the same windows and all subjects. A lag of 6 was chosen, corresponding to the mode of the lag of all windows for all subjects. The second parameter is the embedded dimensions, which depend on the lag. The false nearest neighbor’s method was used to estimate this parameter. For the selected lag or delay (*d* = 6), embedded dimensions of 6 were chosen, corresponding to the mode of the smallest fraction of false neighbors in each window among all subjects. The AMI method is implemented in the R library NonlinearTseries (Garcia and Sawitzki, 2015), whereas the false nearest neighbor’s method is implemented in the R library tseriesChaos (Fabio Di Narzo, 2019). Finally, we estimate the threshold, which defines the minimum threshold for two points considered close or recurrent in the phase space. A general guideline is to select a value that allows the recurrence percentage to be low but not so low as to produce a floor effect (Pellecchia and Shockley, 2005; Webber and Zbilut, 2005). For this, we analyzed different values until we obtained an average recurrence percentage between all windows and subjects that fell around 10%. Our chosen threshold value was 0.02. As for other parameters, the minimum number of points considered as a diagonal or vertical line was 25. Because pupil diameter fluctuations do not usually vary so much in such a short time, this minimum length was chosen to avoid a ceiling effect in the determinism measure or a floor effect in the entropy measure.

From the RQA, we selected three measures to be analyzed between the two groups: recurrence ratio, determinism, and entropy. The recurrence ratio indicates the probability that a specific state will recur. It corresponds to the density of points on a recurrence plot. We expect to see similar recurrences among participants since it was one of the calibration measures for the threshold parameter. However, differences between groups may occur if they suddenly become more or less recurrent for a significant period. Determinism is, in some cases, related to the predictability of the system. It is the percentage of points that form a diagonal line on a recurrence plot. Differences between groups would indicate differences in the variability and predictability of fluctuations. Entropy (Shannon entropy) is related to the complexity of the deterministic structure of the system. It corresponds to the probability that a diagonal line of the recurrence plot has precisely its length. It is a measure related to determinism but gives us more details about the complexity of the variability of the data. For a more detailed description, the interested reader can review the appendix, which contains a mathematical description of the RQA and its measures mentioned here (Supplementary Appendix A).

To evaluate the temporal structure of the pupil diameter fluctuations (i.e., whether it is closer to Brownian or 1/f pink noise), we performed a Power Spectral Density (PSD) using Welch’s method with a sampling frequency of 50hz. This analysis was performed for all subjects in the same windows used in the RQA (500 data points and steps of 100). For pink noise, if plotted on a log–log scale, the logarithmic power function follows a straight line with slope − 1. In contrast, the Brown noise is a line with a slope of −2. White noise has a slope of 0 (Stadnitski, 2012). Thus, the slope of the spectral power was estimated in a log–log scale for each subject and each window. A brief mathematical description of the PSD and the Welch method is described in the appendix (Supplementary Appendix A).

In addition, to explore whether it is possible to detect differences with traditional measures of central tendency, we calculated the group average in each window and the average of the standard deviation of each group and window.

For each measure mentioned (recurrence ratio, determinism, entropy, spectral power density estimate, average per window, and standard deviation per window), a two-way mixed ANOVA was performed, with the measure as the dependent variable, solving and the non-solving group as the between-subjects and the windows as the within-subjects. Post-hoc tests were performed, if appropriate, with the Bonferroni test.

### Performance analysis

One of our study’s primary aims was to replicate prior investigations’ findings using our adapted version of the 8-coin task. This task enabled us to be in line with insight theories. The separate presentation of coins facilitates chunk decomposition, which is impeded when coins are presented together. Additionally, presenting 3D cues, where one coin is placed on top of another and slightly misaligned, promotes constraint relaxation. When coin configurations are presented separately and with 3D cues, it enhances the probability of finding the correct solution. Therefore, our 2-by-2 design should impact the proportion of correct responses, time to complete the task, and the number of attempts made by participants.

Regarding performance, three dependent variables were selected: the task result (the participant solved or did not solve the problem), the total time spent completing the task (in seconds), and the number of attempts before desisting or solving the problem. These variables were analyzed by a 2-by-2 factorial ANCOVA, in which grouping (grouped versus ungrouped) and dimensionality (presence of a 3D cue versus only 2D cue) were the between-subjects factors. The children’s age was selected as a covariate.

## Results

### Performance

The results showed that ungrouped coins led to a higher proportion of correct responses (*M* = 0.515) compared to grouped coins (*M* = 0.224), [*F* (1, 62) = 8.345, *p* = 0.005, η_p_^2^ = 0.12]. Similarly, the use of 3D cues resulted in a higher proportion of correct responses (*M* = 0.476) compared to 2D cues (*M* = 0.263), [*F* (1, 62) = 4.59, *p* = 0.036, η_p_^2^ = 0.07]. However, no interaction effect was observed between grouping and dimensionality [*F* (1, 62) = 1.579; *p* = 0.214].

The analysis also revealed that children took less time to solve the problem with ungrouped coins (*M* = 715.87 s) compared to grouped coins (*M* = 1132.15 s), [*F* (1, 62) = 14.175, *p* = 0.0001, η_p_^2^ = 0.192]. There were no significant effects of dimensionality [*F* (1, 62) = 2.834; *p* = 0.10] or grouping-dimensionality interaction [*F* (1, 62) = 1.5831; *p* = 0.213] on solution time.

The number of attempts was not significantly affected by grouping [*F* (1, 62) = 2.54, *p* = 0.116], dimensionality [*F* (1, 62) = 0.685, *p* = 0.411], or dimensionality-grouping interaction [*F* (1, 62) = 0.348, *p* = 0.558].

**Figure 4:**
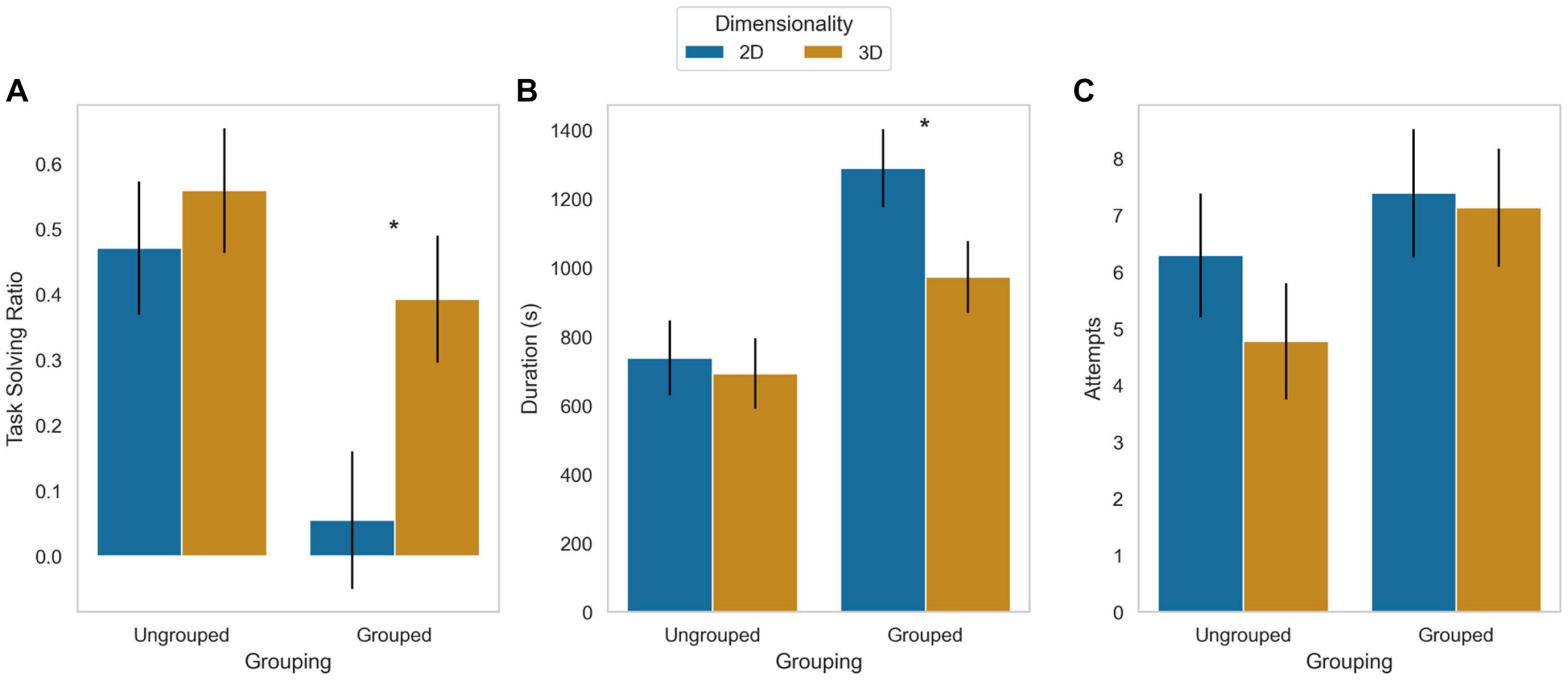
Mean and standard error of task-solving ratio **(A)**, duration or time to complete the task in seconds **(B)**, and the number of attempts **(C)** for each coin grouping condition and each coin dimensionality condition. The age of the participants was controlled as a covariate. Although no significant interaction effects were observed, a subsequent analysis of simple effects, employing the Bonferroni test, revealed that when examining grouped coins, the solving rate was lower, and the duration was longer for 2D cue configurations compared to 3D cues configurations. The asterisk indicates significant differences.

The results showed significant associations between age and three variables: proportion of correct responses [*F* (1, 62) = 14.487, *p* = 0.000, η_p_^2^ = 0.189], solution time [*F* (1, 62) = 4.086, *p* = 0.048, η_p_^2^ = 0.062], and number of attempts [*F* (1, 62) = 4.536, *p* = 0.037, η_p_^2^ = 0.068]. Specifically, as age increased, there was an increase in the proportion of correct responses (*r* = 0.423; *p* = 0.0001) and a decrease in solution time (*r* = −0.264; *p* = 0.031) and the number of attempts (*r* = −0.289; *p* = 0.018). Figure 4 displays the estimated means while controlling for the effect of age.

In sum, even after controlling for age statistically, grouping and dimensionality had independent effects on finding the correct solution and the solution time. Specifically, the proportion of correct responses increased, and solving times decreased when the coins were ungrouped or when 3D cues were utilized. However, no interaction effect was observed between clustering and dimensionality.

Our results provide evidence that our adapted version of the 8-coins task produces results consistent with previous research. Specifically, our findings support the positive impact of using 3D cues and ungrouping the coins on task performance, which were observed not only in the proportion of correct responses but also in the time taken to complete the task and the number of attempts made.

### Pupil diameter fluctuations

Two groups of children were formed depending on who solved the task. Forty-three children did not solve the task (64.2%) and spent an average of 20 min and 24 s (SD = ±5 min approximately) trying to find the solution until they gave up. In contrast, 24 children solved the task (35.8%), spending an average of 6 min and 22 s (SD = ±5 min approximately) to find the solution.

**Figure 5:**
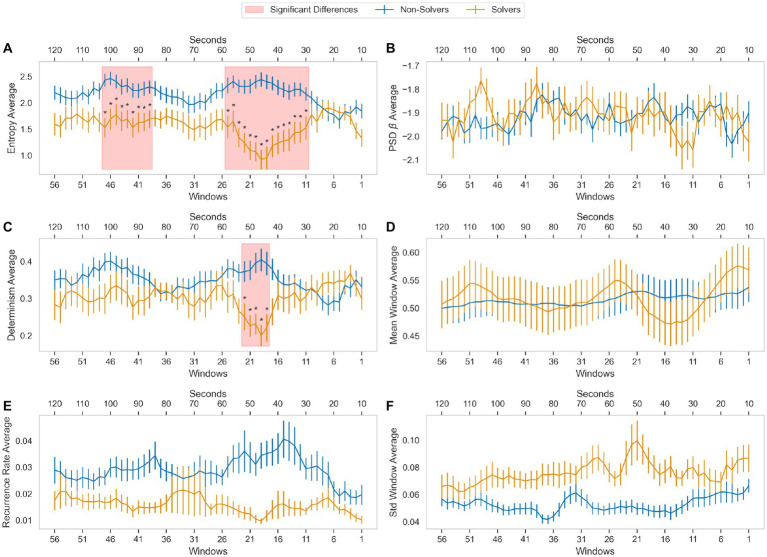
Comparisons of the last 120 s of those who did and did not solve the task on different metrics. These metrics were computed in overlapping windows of 500 data points, moving 100 data points at each step. **(A)** Shows the average entropy by group and window calculated in the RQA. **(C,E)** Shows the average determinism and recurrence rate (RQA metrics). **(B)** Shows the average Power Spectral Density slope estimate for each group in each window. **(D)** Shows the average per group of normalized pupillary diameter fluctuations in each window. **(F)** Shows the standard deviation by group and window. The vertical lines represent the standard error.

We calculated four measures, Entropy (ENT), recurrence ratio (RR), determinism (DET), and Beta scaling exponent (β), for each participant in the last 56 windows, each lasting around 10 s, with an 8-s overlap with the next window. A mixed ANCOVA was used to analyze the data, with the group solvers versus no-solvers being the between-subjects factor and the 56 windows being the within-subjects factor. The model included children’s age as a covariate (refer to Figure 5).

Regarding ENT (Figure 5, Panel A), the solver group had a lower average than the non-solver group [*F* (1, 64) = 10.271; *p* = 0.002; η_p_^2^ = 0.138]. There was also a significant group-windows interaction effect [*F* (55, 3,520) = 2.960; *p* = 0.0000; η_p_^2^ = 0.044], indicating that children who solved the problem had lower ENT than those who did not, specifically in 24 windows, from 47 to 39 and from 25 to 11. The analysis did not reveal any effect of children’s age on ENT [*F* (1, 64) = 1.068; *p* = 0.305] or windows [*F* (55, 3,520) = 1.064; *p* = 0.349] or any significant windows-age interaction [*F* (55, 3,520) = 1.135, *p* = 0.232]. These findings suggest that the amount of uncertainty or randomness in the physiological signal, pupil diameter, increases in the solver group before children find the solution.

In terms of DET (Figure 5, Panel C), only a significant group-windows interaction effect was detected [*F* (55, 3,520) = 1.959; *p* = 0.000; η_p_^2^ = 0.030], indicating that the solver group had lower DET than the not-solver group in 5 windows, from window 22 to 18. The analysis also revealed that children’s age was associated with DET [*F* (1, 64) = 5.27, *p* = 0.025]. However, the analysis did not find any significant effects of groups [*F* (1, 64) = 1.031, *p* = 0.314], windows [*F* (55, 3,520) = 0.701, *p* = 0.954], or windows-age interaction [*F* (55, 53,520) = 0.716, *p* = 0.943].

In terms of RR (Figure 5, Panel E), the solver group had a lower average than the non-solver group [*F* (1, 64) = 4.227; *p* = 0.044; η_p_^2^ = 0.062]. There were no differences among windows [*F* (55, 3,520) = 0.619, *p* = 0.988], and no significant windows-groups or windows-age interaction effects [*F* (55, 3,520) = 1.201, *p* = 0.148 and *F* (55, 3,520) = 0.588, *p* = 0.993, respectively]. Children’s age had no significant effect on RR [*F* (1, 64) = 1.219, *p* = 0.274].

In Figure 5, Panel B, we examined the β scaling exponent and found that there were no significant effects related to children’s age [*F* (1, 64) = 3.280; *p* = 0.075], group [*F* (1, 64) = 0.29, *p* = 0.592], windows [*F* (55, 3,520) = 0.709, *p* = 0.949], windows-group interaction [*F* (55, 3,520) = 1.148, *p* = 0.213], or windows-age interaction [*F* (1, 64) = 0.756, *p* = 0.908]. Furthermore, it is worth noting that the beta values observed in each window fell within a range of −1.7 to −2.0, close to the range associated with Brownian noise.

We also investigated whether nonlinear analysis can detect changes that traditional measures, such as the mean and standard deviation, are unable to detect. In Figure 5, Panels D and F, we analyzed the mean average and the average standard deviation of pupil diameter, respectively. For the mean average (Figure 5, Panel F), we found no interaction effect [*Fs* (55, 3,520) ≤ 1.215; *ps* ≥ 0.135], and there were no significant differences among windows [*F* (55, 3,520) = 0.622, *p* = 0.987] or between groups [*F* (1, 64) = 0.008, *p* = 0.931]. The children’s age, as a covariate, had no effect [*F* (1, 64) = 0.129, *p* = 0.720]. Regarding the average standard deviation of pupil diameter, we found that the solver group had a higher average than the non-solver group [*F* (1, 64) = 26.104; *p* = 0.000; η_p_^2^ = 0.290]. However, no interaction effects were observed [*Fs* (55, 3,520) ≤ 1.224; *ps* ≥ 0.125], and there were no significant differences among windows [*F* (55, 3,520) = 0.686, *p* = 0.963]. The children’s age, as a covariate, had no effect [*F* (1, 64) = 1.038, *p* = 0.312].

The findings suggest that the children who successfully solved the problem displayed higher entropy in their pupillary diameter fluctuations than those who did not. This difference was observed between 92 and 20 s before the end. The determinism also detected group differences between 86 and 76 s before the end. Conversely, RR, β scaling exponent, average standard deviation, and average mean of pupil diameter fluctuations did not differentiate the groups at any time.

In summary, the study analyzed the pupillary diameter fluctuations of children solving an insight problem-solving task. It divided them into two groups: children who solved the task (solver group) and those who did not (non-solver group). The analysis was focused on four nonlinear dynamics measures: Entropy (ENT), recurrence ratio (RR), determinism (DET), and β scaling exponent. The study found that seconds before finding the solution, the solver group had a lower average of ENT and DET than the non-solver group. These results indicate that the solver group had more uncertainty and less predictability than the non-solver group. The results suggest that RQA measures can detect changes that means and standard deviations fail to detect. Finally, two findings are noteworthy regarding the β scaling exponent. Firstly, it failed to detect any significant differences between the solver group and the non-solvers group. Secondly, the values of the said exponent were closer to Brownian noise than to 1/f pink noise.

## Discussion

The main goal of our study was to test if pupillary diameter fluctuations could distinguish between children who successfully solved the adapted 8-coins insight task and those who did not, using analyses associated with dynamical systems. Our key finding was that before solving the task, the pupils of the children who successfully solved the problem showed significant differences. In terms of entropy, such differences appeared between 102 and 86 s, and in between 58 and 30 s. In terms of determinism these differences were seen between 52 and 44 s compared to the unsuccessful group. After this period, pupil fluctuations between the two groups became similar again. Moreover, the recurrence ratio measure revealed that the pupillary diameter fluctuations of children who successfully solved the task had a lower probability of recurrence during all the measure trajectory than those who did not solve the task.

In contrast, the beta exponent of the PSD did not show any differences between the two groups. Additionally, the estimated exponents for each of the 56 windows indicated that the values fluctuated within the range of what is known as Brownian Noise. It is worth noting that when comparing both groups using conventional statistics (mean and standard deviation), we did not find apparent differences as we did with RQA measures (DET and ENT).

During the 8-coin task, each trial involves the child observing and manipulating coins on a surface to find the correct solution. These action-perception chains initially fail and introduce uncertainty into the system, which cannot be dissipated using the system’s existing structures. Consequently, the system is compelled to reorganize itself and find a new structure that can handle the uncertainty more efficiently. From this perspective, solving an insight problem reflects the emergence of a new structure capable of managing a high degree of uncertainty. Our results support this view, as the pupil diameter fluctuations associated with cognitive functioning, as suggested by Hess and Polt (1960, 1964) and Mathôt (2018), were linked to changes in entropy.

However, the most important finding occurs between seconds 58 and 30 (Windows 25 and 11), where an entropy peak was appreciable in the group that solved the problem. During that time segment, the pupil of this group exhibited greater randomness, which was accompanied by less predictability in the measure put forward by Dixon and Bangert (2002), Dixon and Dohn (2003), Stephen et al. (2009), Stephen and Dixon (2009), and Dixon et al. (2010). They proposed that changes in entropy are related to self-organization within the system and reflect a transition from one state to another (Kugler and Turvey, 1988). In the case of insight, the self-organization of a new cognitive structure leads to its emergence. External factors can introduce instability into a system, causing an increase in entropy. As entropy increases, the interaction between perceptual and motor mechanisms promotes adopting a new state that can cope with instability and uncertainty and restore order (Bak, 1996; Jensen, 1998). Thus, the emergence of insight depends on the continuous interaction of various motor and perceptual components with contextual elements and aspects of the 8-coin task. A new pattern or structure emerges at a certain level of disorder, making it possible to solve the task.

Regarding the PSD per window, the Beta scaling exponent (β) of the solvers and the non-solvers groups did not differ throughout the windows. In fact, β scaling exponent of both groups moved systematically in the Brownian noise spectrum, far from 1/f pink noise (Gilden et al., 1995). This kind of noise has been previously discovered in pupil diameter fluctuations (Moon et al., 2014; Kim and Yang, 2017).

Even when 1/f pink noise has been detected in different types of tasks such as visual attention (Kello et al., 2007), time estimation (Gilden et al., 1995; Holden, 2005), rhythmical aiming (Wijnants et al., 2012a,b), automatic response (Gilden, 1997; Beltz and Kello, 2006), mental rotation (Gilden, 2001) and problem-solving (Dixon et al., 2010), the interpretation of such noise remains under discussion. Some researchers have argued that fractal patterns close to 1/f-noise in behavioral and physiological responses show that the boundaries of the system become less rigid, allowing various processes to operate at different timescales to exhibit similar patterns of variability for a period of time and eventually giving rise to the formation of a new, organized structure (Bak, 1996; Jensen, 1998; for an alternative interpretation, see Wagenmakers et al., 2004, 2005; Bogartz and Staub, 2012).

After examining the results, a question that arises is why only entropy and determinism allowed us to find differences between groups. Fractal exponents are influenced by the interplay between internal and external constraints in a task (van Orden et al., 2003; Likens et al., 2015). Dixon et al. proposed that the appearance of 1/f noise is linked to agent-environment dynamics (Bak, 1996; Chemero, 2009). These fractal patterns would reflect the healthy functioning of organisms (Hausdorff et al., 1995; Goldberger et al., 2002). Therefore, the fact that both groups exhibited β exponents close to Brownian noise could indicate a short-term coordinated activity between motor and perceptual processes rather than a long-range correlation. Moreover, it could be argued that pupillary diameter fluctuations have a less flexible functioning compared to other variables, such as angular velocity, in which individuals can demonstrate greater flexibility and dexterity as the task is practiced repeatedly.

This study showed that the pupil diameter fluctuations of both groups exhibited Brownian noise, and noticeable fluctuations of entropy and determinism were observed in the successful group. The reason for this discrepancy remains unclear, and it is possible that the 8-coin task induced a different type of variability in pupil diameter fluctuations, which may reflect the unique cognitive demands of this task.

It is worth mentioning that conventional statistics (average and standard deviation) failed to detect significant differences between groups at specific windows. Therefore, the advantages of Recurrence Quantification Analysis (RQA) in these cases are evident (Marwan and Kurths, 2002; Shockley, 2005; Orsucci et al., 2006; Marwan et al., 2007; Bastos and Caiado, 2010; Ngamga et al., 2016). RQA is a powerful tool to identify complexity and temporal structure patterns in time series data, particularly when the underlying dynamics are poorly understood or when traditional statistical methods are not sensitive enough to detect subtle differences. In this sense, our study adds to the growing body of literature highlighting the value of RQA as a complementary approach to conventional statistics in analyzing complex time series data.

Overall, our findings partially support the direction proposed by Dixon and colleagues, suggesting that human beings (in this case, children) may experience the self-organization of a novel and effective response pattern during the resolution of insight tasks. Specifically, our results indicate that participants who successfully solved the task exhibited distinct patterns of entropy and determinism in their pupil diameter fluctuations before finding the solution, consistent with the behavior expected from self-organizing systems.

However, it remains to be determined whether beta scalar exponents can reliably distinguish individuals who successfully solve the task from those who do not. Furthermore, it is unclear whether fractality is a characteristic that applies equally to all participants when solving insight tasks, or if it may vary depending on the specific nature of the problem. These questions warrant further investigation in future studies.

## Data availability statement

The raw data supporting the conclusions of this article will be made available by the authors, without undue reservation.

## Ethics statement

The studies involving human participants were reviewed and approved by the Comité de Etica Científica de la Universidad de Talca. Written informed consent to participate in this study was provided by the participants’ legal guardian/next of kin. Written informed consent was obtained from the individual for the publication of any identifiable image included in this article.

## Author contributions

RDC: study conception and design. SV-P, RDC, and FM-R: experimental design. RDC, SV-P, and DM-B: data collection. RFC, RDC, SV-P, and FM-R: analysis and interpretation of results. SV-P, RFC, DM-B, and RDC: draft manuscript preparation. All authors reviewed the results and approved the final version of the manuscript.

## Conflict of interest

The authors declare that the research was conducted in the absence of any commercial or financial relationships that could be construed as a potential conflict of interest.

## Publisher’s note

All claims expressed in this article are solely those of the authors and do not necessarily represent those of their affiliated organizations, or those of the publisher, the editors and the reviewers. Any product that may be evaluated in this article, or claim that may be made by its manufacturer, is not guaranteed or endorsed by the publisher.
