# Supplementary material for: The nonlinearity of pupil diameter fluctuations in an insight task as criteria for detecting children who solve the problem from those who do not
Source: Front Psychol. 2023 Jun 23;14:1129355. doi: 10.3389/fpsyg.2023.1129355 (PMC10327553; doi:10.3389/fpsyg.2023.1129355)
Supplement: Supplementary file 1 [file Table_1.DOCX]

**Appendix A**

**Recurrence Quantification Analysis (RQA).** RQA is based on procedures for visualizing recurrence patterns in dynamic systems named recurrence plots (RPs). RPs represent the evolution of a dynamic system, which can be visually analyzed qualitatively and subjectively by observing its structure of isolated points and the diagonal, vertical, and horizontal lines. RPs visualize the recurrence of a state at time $i$ at a different time $j$ in a two-dimensional squared matrix with ones and zeros (or black and white dots in a plot), where both are time axes. A RP can be mathematically expressed as:

$$R_{i,j}=\Theta\left( \varepsilon-\parallel\vec{x_{i}}-\vec{x_{j}}\parallel\right), x_{i}\in\mathbb{R}^{m}, i,j=1,\ldots,N,$$

( 1 )

Where $N$ is the number of considered states of $x_{i}$. $\varepsilon$ is a threshold distance, $\parallel\cdot\parallel$ is the neighborhood measure (usually the Euclidean norm), and $\Theta(\cdot)$ is the Heaviside function (a unit step function), which is 1 when $x_{i}\approx x_{j}$, and 0 otherwise. $x_{i}\approx x_{j}$ means equality up to an error or distance threshold $\varepsilon$. In short, the matrix tells us when similar states of the underlying system occur.

RQA can reconstruct the phase space of a multidimensional system based on Taken’s embedding theorem. This theorem proposes that under some conditions, it is possible to roughly reconstruct the state-space of a dynamical system by delay-embedding only one of its time series as follows:

$$\vec{x_{i}} = \left( u_{i}, u_{i+d}, ... , u_{i+d\left( m-1 \right)} \right)$$

( 2 )

Where $u_{i}$ is the time series, $m$ is the embedding dimension, and $d$ is the time delay. The delay parameter helps to retrieve the m-dimensions using the delayed embedding method. This parameter considers the properties of the sample of observations, finding points in the time series that can be used to reconstruct the latent dimensions. The embedded dimensions ($m$) correspond to an estimate of the latent dimensions that would configure the observed system. Finally, the radius (ε) specifies an interval where two values are considered equal.

Several measures can be obtained from the RQA. For this study, we considered three; the recurrence ratio (RR), determinism (DET), and Entropy (ENT). RR is the density of recurrence points in a recurrence plot and indicates the probability that a specific state will recur. RR is estimated as follows:

$$RR=\frac{1}{N^{2}}\sum_{i,j=1}^{N} R(i,j)$$

( 3 )

DET is the percentage of recurrence points that form diagonal lines in the recurrence plot of minimal length $\mathcal{l}_{min}$ (set to 25 for this study.). DET relates to the predictability of the system. DET is estimated as follows:

$$DET=\frac{\sum_{\mathcal{l=}\mathcal{l}_{min}}^{N} \mathcal{l}P\left( \mathcal{l} \right)}{\sum_{\mathcal{l=}1}^{N} \mathcal{l}P\left( \mathcal{l} \right)}$$

( 4 )

ENT is the probability $p\mathcal{(l)}$ that a diagonal line has exact length $\mathcal{l}$. It is the Shannon Entropy that represents the frequency distribution $P\mathcal{(l)}$ of the diagonal line lengths. ENT can be related to the complexity of the deterministic structure in the system. Low entropy represents little complexity in the distribution of periodic components, and high entropy represents diversity in short and long-duration periodicities. The probability $p\mathcal{(l)}$ that a diagonal line has exact length $\mathcal{l}$ and the Shannon Entropy (ENT) can be estimated as follows:

$$p\left( \mathcal{l} \right)=\frac{P\left( \mathcal{l} \right)}{\sum_{\mathcal{l=}\mathcal{l}_{min}}^{N} P\left( \mathcal{l} \right)} ENT= -\sum_{\mathcal{l=}\mathcal{l}_{min}}^{N} p\left( \mathcal{l} \right)\ln p(\mathcal{l})$$

( 5 )

**Power Spectral Density (PSD).** PSD Corresponds to the distribution of the average power of a signal. It shows which frequency variations are strong and which are weak. The unit of PSD is energy (variance) per frequency (width). The power spectral density is just the Fourier transform of the signal. The definition of discrete-time PSD at frequency $f$ via periodogram is:

$$PSD\left( f \right)=\frac{\left( \Delta t \right)^{2}}{T}\left| \sum_{n=1}^{N} x_{n} e^{i2fn\Delta t} \right|^{2}$$

( 6 )

Where T is the total signal or window duration given by $T=(N-1)\Delta t$, where $\Delta t$ is the sample interval.

For this study, we used Welch’s method for the spectral density estimation. Instead of calculating the Fourier transform on the entire time window, it calculates the Fourier transform in overlapping windows and the power spectrum in each window. After calculating all windows, it calculates the average of all power spectrums. Welch’s method gets a smoother power spectrum than the full Fourier transform. Also, Welch’s method is more robust to some non-stationarities.
