# Supplementary material for: The nonlinearity of pupil diameter fluctuations in an insight task as criteria for detecting children who solve the problem from those who do not
Source: Front Psychol. 2023 Jun 23;14:1129355. doi: 10.3389/fpsyg.2023.1129355 (PMC10327553; doi:10.3389/fpsyg.2023.1129355)
Supplement: Supplementary file 2 [file Table_2.docx]

**Appendix B**

A 2-by-2 mixed ANCOVA was conducted. Dimensionality (3D versus 2D), and the grouping (grouped versus ungrouped) were the between-subjects factors and the fifty-six windows were the within-subjects factor. The model included children's age as a covariate (see Table 1).

It was found significant interactions between dimensionality and windows for entropy, determinism and standard deviation. Specifically, at window 25, 3D cues led to higher entropy than 2D cues. At window 17 3D cues resulted in lower average determinism than 2D cues. In terms of standard deviation, there was higher variability for 3D cues than 2D cues between window 25 and 31.

However, we did not observe any significant effects for the recurrence ratio or the mean and beta scaling exponents.

|  | Entropy | | Determinism | | Recurrence Ratio | | Beta Scaling Exponent | | Mean | | Standard Deviation | |
| --- | --- | --- | --- | --- | --- | --- | --- | --- | --- | --- | --- | --- |
| Origen | F | p | F | p | F | p | F | p | F | p | F | p |
| windows | 1.749 | .001 | 1.135 | .231 | 1.003 | .469 | .611 | .989 | .646 | .980 | .818 | .829 |
| windows * age | 1.742 | .001 | 1.097 | .291 | .830 | .810 | .671 | .970 | .755 | .909 | .836 | .800 |
| windows * (G vs. UG) | 1.011 | .452 | .720 | .940 | 1.255 | .099 | 1.245 | .107 | .678 | .967 | .922 | .639 |
| windows * (2D vs 3D) | 1.624 | .003 | 1.849 | .000 | 1.155 | .204 | .963 | .553 | 1.012 | .330 | 1.784 | .000 |
| windows * (G vs. UG) * (2D vs 3D) | .935 | .612 | .482 | 1.000 | 1.068 | .342 | 1.163 | .193 | .528 | .998 | .708 | .949 |
| age | 4.915 | .030 | 7.679 | .007 | 3.129 | .082 | 3.544 | .064 | .221 | .640 | .847 | .361 |
| (G vs. UG) | 2.222 | .141 | .188 | .666 | 1.907 | .172 | .110 | .741 | .151 | .699 | 2.429 | .124 |
| (2D vs 3D) | 1.239 | .270 | .458 | .501 | .084 | .773 | 2.335 | .132 | .144 | .706 | .813 | .371 |
| (G vs. UG) * (2D vs 3D) | .171 | .680 | 2.364 | .129 | .284 | .596 | .003 | .953 | .123 | .726 | .399 | .530 |
